# Estimation of Environmental Effects and Response Time in Gas-Phase Explosives Detection Using Photoluminescence Quenching Method

**DOI:** 10.3390/polym16070908

**Published:** 2024-03-26

**Authors:** Daegwon Noh, Eunsoon Oh

**Affiliations:** 1 Department of Physics, Chungnam National University, 99 Daehakro, Yuseong-gu, Daejeon 34134, Republic of Korea; fo1109@cnu.ac.kr; 2 Institute of Quantum Systems (IQS), Chungnam National University, 99 Daehakro, Yuseong-gu, Daejeon 34134, Republic of Korea

**Keywords:** explosives detection, environmental effects, photoluminescence, conjugated polymer, chemical sensing, fluorescence quenching

## Abstract

Detecting the presence of explosives is important to protect human lives during military conflicts and peacetime. Gas-phase detection of explosives can make use of the change of material properties, which can be sensitive to environmental conditions such as temperature and humidity. This paper describes a remote-controlled automatic shutter method for the environmental impact assessment of photoluminescence (PL) sensors under near-open conditions. Utilizing the remote-sensing method, we obtained environmental effects without being exposed to sensing vapor molecules and explained how PL intensity was influenced by the temperature, humidity, and exposure time. We also developed a theoretical model including the effect of exciton diffusion for PL quenching, which worked well under limited molecular diffusions. Incomplete recovery of PL intensity or the degradation effect was considered as an additional factor in the model.

## 1. Introduction

Detecting the presence of explosives is important to protect human lives in military conflicts and in peacetime. In the case of landmine detection, the predominant approach is the use of metal detectors to identify metallic components of buried mines. However, this method faces challenges when detecting non-metallic mines, such as wooden mines or plastic explosives. To address this, ground penetrating radar (GPR) has been developed. Nevertheless, GPR has drawbacks such as complex signal processing and high power consumption. On the other hand, most explosives contain nitro compounds such as trinitrotoluene (TNT) and dinitrotoluene (DNT), and direct sensing of nitro molecules is possible via ion mobility spectrometry, surface-enhanced Raman spectroscopy, and luminescence/fluorescence methods [1,2,3,4,5,6,7,8,9,10,11,12,13].

For fluorescence methods, various materials, including conjugated polymers (CP), inorganic materials, dendrimers, aggregation-induced emission materials, etc., have been investigated [14,15,16,17,18,19]. Although various methods, including fluorescence quenching, fluorescence turn-on, and spectral shift, have been investigated, fluorescence quenching of CP has been most extensively studied. In this paper, rather than fluorescence, we use the more general term of photoluminescence (PL). In case of the PL quenching method, photoelectron transfer is known to play a major role; i.e., the LUMO level of a sensing material lies higher than those of nitro-containing molecules, and photoelectrons in the LUMO level transfer to the target molecules, allowing for the non-radiative recombination of electrons or the decrease in PL intensity.

For real-time sensing, non-invasive and passive explosives detection in the vapor phase usually offers more advantages. Our motivation for the PL quenching study is utilizing CP films for real-time applications. In many cases, CP may undergo film deterioration, and its intensity may change over time. Moreover, if explosive molecules diffuse into a film, some molecules may not diffuse out of the CP film for a long time, making continuous detection difficult. Even though the desorption of target molecules may be improved at higher temperatures [20], special care is necessary because organic materials are generally unstable at high temperatures, especially under light exposure. Explosives detection studies to date have focused on sensitivity and selectivity, but careful investigations regarding how long the films can be used continuously, limiting climatic conditions for device operation, and the compensations required for PL intensity are all very important. Among the various CP materials used today, the use of some CP films may make long-term detection difficult due to film deterioration.

Ensuring a low false-positive rate under real environmental conditions has been a challenge in PL quenching-based sensors [21] as well as in other sensors making use of the electrical conductivity. In this regard, improving the environmental stability of PL intensity is necessary. For example, false positive alarms may be caused by the reduced PL intensity simply due to hot air inside of a building as we try to detect bombs in the building. Thus, to improve the reliability of the PL quenching sensors, there must be extra efforts to fully understand the changes in the PL intensity caused by various environmental conditions. The ultimate goal of our research is to develop an algorithm to compensate for the effects of environmental conditions. One alternative way is to keep the sensing parts at constant temperature and humidity, etc., which would also be difficult due to the continuous flow of the air that needs to be tested.

This paper demonstrated how one can evaluate environmental impacts due to temperature and humidity in explosives detection using PL quenching methods. PL quenching (PQ) efficiencies were measured repeatedly with short-time exposure of the explosive vapors under near-open conditions, while temperature and humidity inside of the PL apparatus were recorded in real time.

## 2. Experimental Set-Up for Data Acquisition

### 2.1. Materials and Methods

Quartz substrates were first wiped with ethanol, and then the substrates were sonicated for 10 min each in acetone, in isopropyl alcohol, and in ethanol. Quartz substrates were preferred to glass for reduced UV absorption and luminescence. When considering stability issues, pentiptycene-containing conjugated polymer (PCC) and PEE films were found to be superior to MEH-PPV or PDY-132 (super yellow) thin films under our experimental conditions, and PCC was used for sensing material. In Supplement Figure S1, we compared the PL intensity over time for various polymer films. PEE, MEH-PPV, and PDY-132 powders were purchased from Sigma-Aldrich (St. Louis, MO, USA). Using the PCC powder provided by PNL global (Seoul, Republic of Korea), the PCC solution was prepared with toluene solvent. Then, PCC films were obtained by spin-coating with 2000 rpm (revolutions per minute) for 60 s on the cleaned quartz substrates. After the spin-coating, the films were thermally annealed on a hot plate for ~1 min at 100 °C in order to ensure the complete evaporation of toluene. The thickness of the film was estimated to be ~20 nm.

Although TNT is more commonly used for explosives, 2,4-dinitrotoluene (2,4-DNT) is one of the most prevalent in the vapor phase chemical signature of landmines [22]. In addition, since the vapor pressure of DNT is significantly higher than that of TNT, we used 2,4-DNT for vapor explosive detection.

PL measurements were performed in two different experimental setups; a home-made PL system that we reported previously [11] and a detector system developed by PNL Global. The former was used to measure PL spectra and to separately monitor temperature and humidity effects. The latter was used to detect 2,4-DNT vapor under near-open conditions in a custom-built lab.

Most of the vapor measurements were performed without controlling the room condition except for the air circulation system. However, some measurements were performed with a radiator-type heater (for a room) and a plate-type heater (for a box).

### 2.2. Automatic Shutter Control and Real-Time PL Intensity Monitoring System

**Figure 1:**
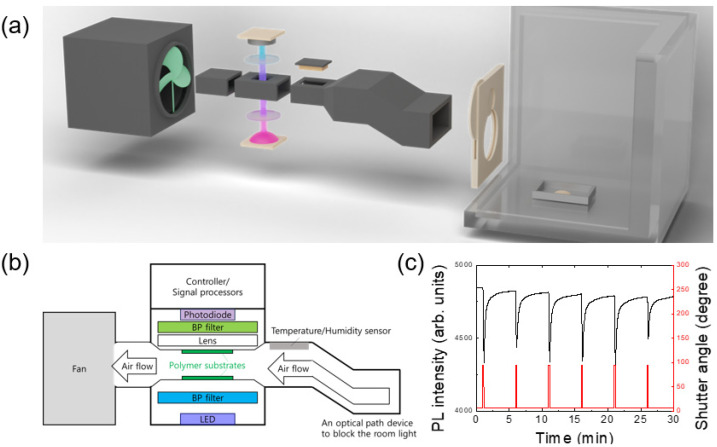
(**a**) A schematic picture of the experimental set-up consisting of a box with a shutter and a PL apparatus. A total of 0.1 g of 2,4-DNT powder was placed in a stainless steel bowl. (**b**) A schematic diagram of the PL apparatus. (**c**) Photoluminescence response by shutter opening and closing. The period was 5 min and the shutter opening time was 10 s.

A shutter was installed on an acrylic box (20 × 20 × 40 cm^3^), and the PL intensity of polymer film inside of the equipment placed in front of the shutter was monitored by opening and closing at regular times (Figure 1). Only 0.1 g of 2,4-DNT powders were placed inside an acrylic box with a shutter that was remotely controlled in another city so that the exposure to the potentially harmful vapors could be minimized.

Data communications between the equipment and a computer were carried out through Bluetooth wireless communication, and the shutter was directly controlled from the computer through wired serial communication. With pre-scheduled experimental plans, measurements (the equipment control, the shutter operation, and data transmission) were started by remotely accessing the control computer (located in Seoul) from another computer (located in Daejeon), and data were typically acquired for several hours. 

Figure 1a shows a schematic diagram of a box with an automatic shutter control system as well as an apparatus used for PL quenching measurements. The temporal variation of PL intensity (black) together with that of the shutter angle (red) are shown in Figure 1c. The apparatus contains sensing polymer substrates, an LED, a photo-diode, a fan, and a flow-guiding part to supply air into an optical sensing part as well as to completely block the room light (Figure 1b). In this study, air flow velocity near the polymer sample was ~10 m/s.

As seen in Figure 1c, PL intensity was immediately decreased within 1 s of the shutter opening and started recovering within 2 s of the shutter closing, although the complete recovery of PL intensity may take a few minutes. The time response is discussed in Section 3.2. 

To monitor the PL intensity, an excitation wavelength of 400 nm from a light-emitting diode (LED) was used. For real-time intensity monitoring for gas-phase sensing, a photo-diode was utilized in the PL apparatus, and band-pass (BP) optical filters were used to remove the parasitically scattered light from the LED. 

### 2.3. Measurements of Photoluminescence Spectra

**Figure 2:**
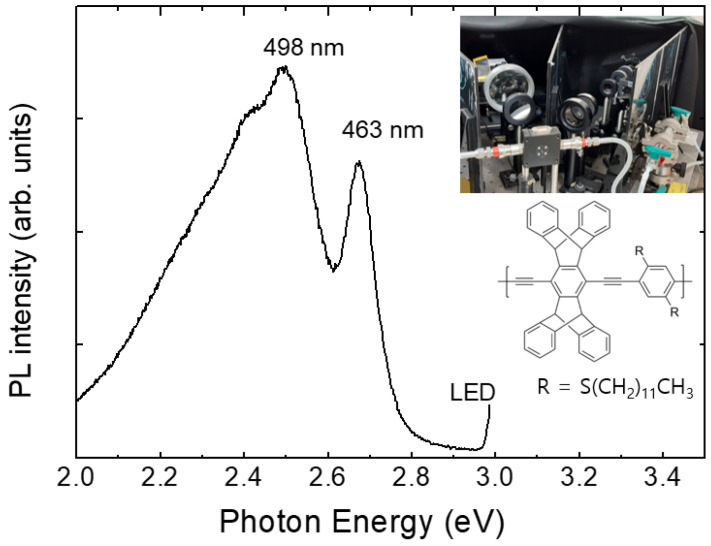
Photoluminescence (PL) spectra of a PCC polymer film at room temperature. A picture of a home-made PL system is included.

Pentiptycene-containing conjugated (PCC) polymer films coated on quartz substrates were used for explosives gas sensing. Figure 2 shows the PL spectrum of a PCC film. In the set-up, the parasitically scattered light at 400 nm (3 eV) was monitored to ensure the absence of water condensation in the optical components as well as to ensure the stability of the excitation light.

### 2.4. In Situ Measurement of Temperature and Humidity

**Figure 3:**
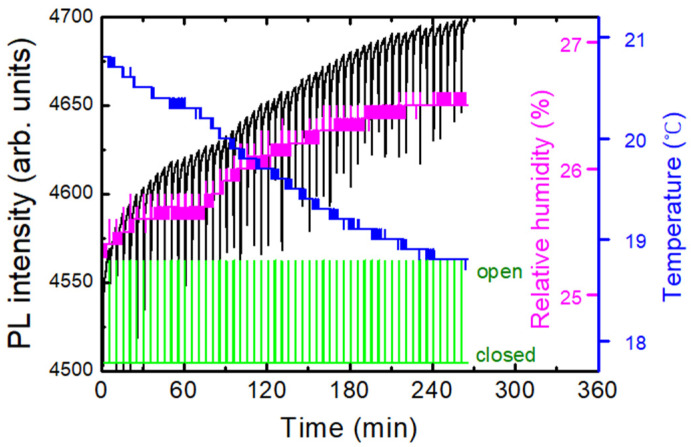
An example of real-time PL intensity (black), temperature (blue), and relative humidity (magenta) monitored inside of a PL quenching apparatus. Also shown is the shutter angle (green). PL responses for the exposure of DNT vapors are observed in 5-min intervals.

Temperature and humidity sensors were positioned close to the polymer substrates inside of the apparatus. Figure 3 shows an example of a dataset composed of temperature, humidity, PL intensity, and shutter angle as a function of time. With the decrease in the temperature (blue), relative humidity (magenta) was increased as expected, and PL (black) quenching was observed for the exposure of DNT vapors as a response to the opening of the shutter in five-minute intervals. It was often observed that the PQ was decreased after 20–30 times of shutter openings, as the vapor molecules repeatedly escaped from the box. On the other hand, the repeated responses demonstrate that the amount of vapor molecules that escape during 10 s is considerably lower than the total amount of the DNT vapor molecules inside the box.

## 3. Results and Discussion

Most measurements were started in the evening, and the air of the lab was ventilated the next morning by opening the windows. After using a polymer film over one month, the PL intensity gradually decreased, but the sensing performance was not noticeably decreased until the PL intensity became about 1/3 of the initial value. Even when no measurements were carried out for 10 days, the PL intensity was found to be slightly decreased. For this reason, spin-coated polymer films were usually stored in a vacuum-sealed box and were covered with aluminum foil to block the lights.

### 3.1. Shutter Exposure Time Dependence

**Figure 4:**
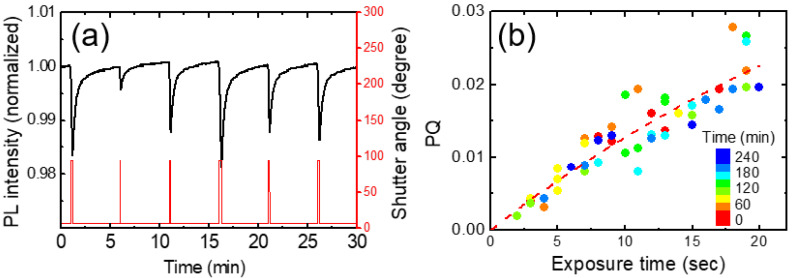
(**a**) PL quenching vs. time for PCC polymer film. (**b**) The corresponding PL quenching (PQ) as a function of the exposure time, which was obtained from the raw data as in (**a**). The dashed curve is a fitting curve obtained from a theoretical model used for response time analysis.

PL intensity was decreased whenever a shutter was open, as DNT molecules were adsorbed on the surface of a polymer film, or possibly diffused into the film. Figure 4a shows the integrated PL intensity (black) of a polymer film and shutter angle (red) as a function of time with various opening (exposure) times. The change of the PL intensity every 5 min with the shutter opening was apparent in Figure 4a. The exposure time for the DNT vapor was pre-scheduled with random values ranging from 2 s to 20 s.

The time response of the PL intensity with the opening and closing of the shutters will be discussed in detail in the next section together with a theoretical model. 

### 3.2. Response Time Analysis

In this section, we discuss the temporal PL behavior during quenching and recovering responses. We started with the classical Langmuir adsorption model for the analyte mass transport and utilized the exciton diffusion model for quenching efficiency calculation. 

In some cases, the dynamics of analyte molecules in PL quenching were explained by molecular diffusion into sensing films. After the sensing films were exposed to quencher vapors, swelling and mass increase in the polymer films were reported through neutron scattering and in situ QCM measurements, which supported a specific diffusion mechanism of analyte molecules, the so-called case 2, or super case 2 [23,24,25,26]. In addition, as a result of these studies, it was argued that molecular diffusion may be more important than exciton diffusion [25], but most of these diffusion studies have been conducted under conditions such as exposure to analyte vapor for several minutes or longer. In our cases with repeated exposure to diluted analyte(2,4-DNT) vapor, it is unclear how much exciton diffusion and analyte diffusion contribute to quenching, respectively, and a cautious approach may be necessary.

Regardless of the molecular diffusion and adsorption mechanism, desorption of adsorbed/absorbed molecules does not ensure complete recovery of PL intensity. We note here that in most polymer/dendrimer PL quenching sensors, the PL intensity of a film once exposed to the DNT analytes may not recover to its original value despite the decrease in adsorption/absorption mass [25,26]. This phenomenon is probably related to the binding affinity between the analyte molecules and the polymers, as well as the molecular diffusion behavior in the film, and the quenching efficiency may also be affected by the binding affinity [23].

To obtain a sufficient amount of data without significant DNT diffusion into the sensing film, we chose a rather short exposure time (10 s) and a time interval of 5-min. We try to understand the temporal response of PL and environmental effects on PL under such simple conditions first. Then, an incomplete recovery of the PL, which was more pronounced for higher DNT vapor pressures and longer DNT exposures, will be discussed in Section 3.4. 

We start with the classical Langmuir adsorption model to explain the molecular dynamics of the analyte vapor molecules. In the model, the adsorption and desorption processes can be expressed by the following equations:

Adsorption:

$$\theta_{0}=0,$$

$$\frac{d\theta }{dt}=k_{adsorption}P\left(1-\theta \right)-k_{desorption}\theta ,$$

$$\theta_{\left(t\right)}=\frac{K_{eq}P}{1+K_{eq}P}\left(1-e^{-k_{desorption}\left(K_{eq}P+1\right)t}\right).$$

Desorption:

$$\frac{d\theta }{dt}=-k_{desorption}\theta ,$$

$$\theta_{\left(t\right)}=\theta_{q0}e^{-k_{desorption}t},$$

where

$$\begin{array}{c} t:time,\\ P:fractional\;pressure\;of\;the\;quencher\;vapor\;on\;the\;surface\;of\;films,\\ \theta :surface\;coverage,\\ \theta_{q0}:surface\;coverage\;at\;the\;moment\;when\;the\;shutter\;is\;closed\;\left(t=t_{q}\right),\\ k_{adsorption}:adsorption\;rate\;constant,\\ k_{desorption}:desorption\;rate\;constant,\\ K_{eq}=\frac{k_{adsorption}}{k_{desorption}}. \end{array}$$

To relate the surface coverage and the quenching efficiency, we make use of a 1D exciton diffusion model.

If we consider only the quenching sites at the surface (*x* = *d*), the differential equation for the exciton diffusion model will have the following form:

for 0<x<d,

$$\frac{\partial n_{\left(x,\;t\right)}}{\partial t}=D\frac{\partial^{2}n_{\left(x,\;t\right)}}{\partial x^{2}}-\frac{1}{\tau }n_{\left(x,\;t\right)}+g.$$

With the following boundary conditions:

$$\frac{\partial n_{\left(x,\;t\right)}}{\partial x}{|}_{x=0}=0,$$

$$\frac{\partial n_{\left(x,\;t\right)}}{\partial t}{|}_{x=d}=D\frac{\partial^{2}n_{\left(x,\;t\right)}}{\partial x^{2}}{|}_{x=d}+k_{q}n_{\left(d,\;t\right)}\theta_{\left(t\right)}.$$

At the surface (x=d), the amount of exciton diffusion flux is proportional to the rate of the quenching reaction.

Here,

$$\begin{array}{c} x:the\;coordinate\;which\;is\;normal\;to\;the\;film\;surface\\ n:exciton\;density\\ \tau :exciton\;lifetime\\ g:exciton\;generation\;rate\\ D:exciton\;diffusion\;constant\\ d:film\;thickness\\ k_{q}:quenching\;rate\;constant\;when\;\theta =1 \end{array}$$

The solution of this differential equation is

$$n_{\left(x,\;t\right)}=\tau g\left(1-\frac{\cosh\left(\frac{x}{L_{D}}\right)\tau k_{q}\theta_{\left(t\right)}}{\left(1+\tau k_{q}\theta_{\left(t\right)}\right)\cosh\left(\frac{d}{L_{D}}\right)}\right),$$

$$n_{\left(d,\;t\right)}=\frac{\tau g}{1+\tau k_{q}\theta_{\left(t\right)}}.$$

where

$$L_{D}:exciton\;diffusion\;length\;\left(L_{D}=\sqrt{D\tau }\right).$$

Then, PL intensity *I*_(*t*)_ becomes:

$$\begin{array}{cc} \frac{I_{\left(t\right)}}{I_{0}}=\frac{\int_{0}^{d}n\;dx}{\int_{0}^{d}n{|}_{\theta =0}\;dx} & =\frac{\int_{0}^{d}\left(1-\frac{\cosh\left(\frac{x}{L_{D}}\right)\tau k_{q}\theta_{\left(t\right)}}{\left(1+\tau k_{q}\theta_{\left(t\right)}\right)\cosh\left(\frac{d}{L_{D}}\right)}\right)\;dx}{\int_{0}^{d}1\;dx},\\ & =1-\frac{\tau k_{q}\theta_{\left(t\right)}}{1+\tau k_{q}\theta_{\left(t\right)}}\frac{L_{D}}{d}tanh\left(\frac{d}{L_{D}}\right). \end{array}$$

If we combine the exciton diffusion model with the Langmuir adsorption model, the PL intensity is as follows:

Adsorption:

$$\frac{I_{\left(t\right)}}{I_{0}}=1-\frac{\tau k_{q}\frac{K_{eq}P}{1+K_{eq}P}\left(1-e^{-k_{desorption}\left(K_{eq}P+1\right)t}\right)}{1+\tau k_{q}\frac{K_{eq}P}{1+K_{eq}P}\left(1-e^{-k_{desorption}\left(K_{eq}P+1\right)t}\right)}\frac{L_{D}}{d}tanh\left(\frac{d}{L_{D}}\right).$$

Desorption:

$$\frac{I_{\left(t\right)}}{I_{0}}=1-\frac{\tau k_{q}\theta_{q0}e^{-k_{desorption}\left(t-t_{q}\right)}}{1+\tau k_{q}\theta_{q0}e^{-k_{\mathrm{desorption}}\left(t-t_{q}\right)}}\frac{L_{D}}{d}tanh\left(\frac{d}{L_{D}}\right),$$

$$\begin{array}{c} \theta_{q0}:surface\;coverage\;at\;t=t_{q},\\ t_{q}:exposure\;time. \end{array}$$

Then, the expression for *PQ* in adsorption becomes the following:

$$PQ=\frac{\left(1-e^{-kt}\right)}{Ak+\left(1-e^{-kt}\right)}\Phi ,$$

where

$$A=\frac{1}{\tau k_{q}k_{adsorption}P},$$

$$k=k_{desorption}\left(K_{eq}P+1\right),$$

$$\Phi =\frac{L_{D}}{d}tanh\left(\frac{d}{L_{D}}\right).$$

The fitting parameter Φ represents the effect of polymer film thickness and exciton diffusion length, which is independent of other parameters such as k and P. For photovoltaic conversion in organic photovoltaics, exciton diffusion is important since excitons need to be collected before the recombination of excitons [27]. In the case of PL quenching for sensing applications, *PQ* will be affected by the exciton diffusion since the photoelectrons that are generated within the diffusion length from the film surface can diffuse to the surface where the sensing molecules are adsorbed.

In these expressions, some effects were not taken into consideration, such as the photo-induced PL enhancement, photo-degradation, and diffusion (out-diffusion) of DNT molecules into (from) the inner part of the polymer films.

To take care of these effects, we modified the equations as follows: 

For adsorption:

$$\frac{I_{\left(t\right)}}{I_{0}}=1-\frac{\left(1-e^{-kt}\right)}{Ak+\left(1-e^{-kt}\right)}\Phi +bt,$$

$${PQ}_{\left(t\right)}=1-\frac{I_{\left(t\right)}}{I_{0}}=\frac{\left(1-e^{-kt}\right)}{Ak+\left(1-e^{-kt}\right)}\Phi -bt.$$

For desorption:

$$\begin{array}{c} \frac{I_{\left(t\right)}}{I_{0}}=1-a\frac{\left(1-e^{-kt_{q}}\right)e^{-k_{desorption}\left(t-t_{q}\right)}}{Ak+\left(1-e^{-kt_{q}}\right)e^{-k_{desorption}\left(t-t_{q}\right)}}\Phi -\left(1-a\right)\frac{\left(1-e^{-kt_{q}}\right)}{Ak+\left(1-e^{-kt_{q}}\right)}\Phi +bt,\\ =1-a\frac{\left(1-e^{-kt_{q}}\right)e^{-k_{desorption}(t-t_{q})}}{Ak+\left(1-e^{-kt_{q}}\right)e^{-k_{desorption}(t-t_{q})}}\Phi -\left(1-a\right)\left({PQ}_{\left(t_{q}\right)}+bt_{q}\right)+bt,\\ a,\;b:constants. \end{array}$$

Here, the variable “*a*” represents PL intensity recovered during the refresh period with respect to *I*_0_. If PL is completely recovered, *a* = 1. A non-zero value of 1 − *a* means that the DNT molecules are strongly bound at the surface, or the DNT molecules from the inner part of the polymer films do not diffuse out completely. The parameter “*b*” represents a slow linear term, which may be due to other temporal responses including photo-induced PL enhancement and photo-degradation, regardless of vapor exposure.

**Figure 5:**
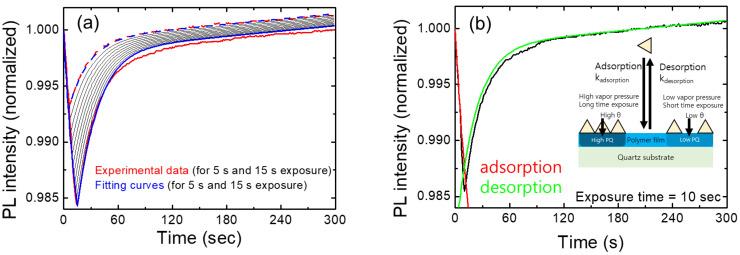
(**a**) PL intensity for various exposure times as a function of time. The shutter opening time is set to zero. The PL values were averaged for each exposure time. Theoretical curves are also shown for comparison. The dash and solid curves represent 5 s and 15 s results, respectively. Black curves show the expected PL intensities with exposure times ranging from 6 s to 14 s. (**b**) PL intensity variation as a function of time (black). Fitting curves of the PL intensity using a theoretical model are shown in red and green. Fitting parameters are listed in Table 1.

In Figure 5a, we show the temporal behavior of normalized PL intensities for various exposure times. The red curves represent experimental results for exposures of 5 s and 15 s, while the blue curves depict the model-predicted PL intensities for the corresponding exposure times. Additionally, black curves illustrate the expected PL intensities at 1 s intervals, ranging from 6 s to 14 s. These calculations were performed using fitting parameters derived from the 10 s exposure data. 

As we checked the value of the linear term of bt in Table 1, the linear contribution was significantly smaller than the other two terms, as expected. Despite many assumptions and simplifications, this model seemed to well explain the experimental results, at least under the conditions of short exposure times (up to 15 s) and for relatively low vapor concentrations. 

Table 1 shows the fitting parameters used for Figure 5. Instead of the individual values of “k_adsorption_”, “k_q_”, and dilution factor “P_r_”, the value of “τk_q_k_adsorption_P_2,4-DNT_P_r_” represents the quenching speed. P_2,4-DNT_ is the equilibrium vapor pressure of DNT and the dilation factor “P_r_” is to take care of the fact that the vapor pressure of DNT molecules is significantly lower than that of the equilibrium vapor pressure at a given temperature due to the small amount of powders inside of the box; another factor for the dilation of the DNT vapors was due to the mixture of the air from the box and the air in the lab.

As the exposure time and the vapor pressure increase, the degradation of the polymer film may become severe; hence, it will be difficult to describe the temporal behavior of the PL intensity with this simplified model. Swelling and mass increase in polymer thin films exposed to high concentrations of quencher molecules have been reported.

In this study, the surface adsorption by exposure to low vapor pressure for a short time and the desorption of the adsorbed molecules were our main concerns, and the experiment was conducted under the condition that PL intensity was restored by more than 80% after the five-minute refresh time; the effect of molecular diffusion into the thin film was not our primary factor. 

To explain the change in PL intensity due to continuous excitation light illumination and the incomplete recovery of the PL intensity in the quenching reaction, a and b parameters were introduced into the equation for the fitting process. 

### 3.3. Effect of Temperature and Humidity

In this section, we discuss the temperature and humidity dependence of the polymer PL intensity and the real-time variation of the PL intensity, rather than of the quantitative characteristics of quenching behaviors.

It is well-known that PL intensity decreases with increasing temperature since the lattice vibrations, or phonons induce non-radiative recombination of photoexcited electrons. Besides PL intensity, other properties such as PL spectra [28], the adsorption of target molecules, and the efficiency of the quenching reaction can also be affected by temperature; thus, great efforts are needed to resolve all these issues. Of course, temperature has a great impact on the vapor pressure of target molecules, which will be discussed shortly at the end of this paper.

Although various temperature-dependent properties can affect sensing, PL intensity change with temperature is particularly important and also easy to monitor because the intensity changes even without the presence of analyte vapor. On the other hand, in the case of adsorption or reaction rate change, it is difficult to distinguish whether the effect is due to the vapor pressure or due to the reaction rate, and the temperature dependence of the reaction rate is not considered in this study.

In general, reducing the effect of humidity is an important issue for chemical sensors. In many cases, water molecules can induce oxidation or degradation of materials, and they can cause variations in the PL intensity for many materials including perovskites and polymers [29,30]. To prevent this, an additional hydrophobic protective layer or functionalization can be introduced [31], but the change due to moisture is still an important factor because such a protective layer can affect the sensing characteristics.

The optical properties of polymers are influenced by various factors such as molecular conformations, aggregation, and degree of crystallization [32,33]; the infiltration of water molecules into sensing materials can have a significant impact on those characteristics. One extreme example is a hydrogel, wherein its structure can be affected by humidity [34]. Not only for hydrophilic polymers but also for hydrophobic polymers, it is important to consider the influence of humidity on PL intensity.

**Figure 6:**
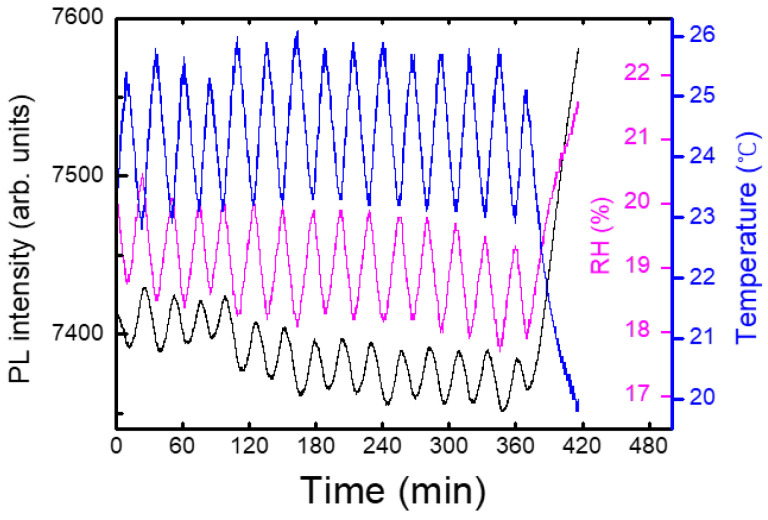
As a heater inside the lab produced oscillations of temperature (blue), relative humidity (magenta) and PL intensity (black) were correspondingly changed.

On the contrary to most DNT sensing systems in a lab, our experiments were carried out in a near-open system, where the air from the box was mixed with the air in the lab. Figure 6 shows an example of a temporal behavior of PL intensity (black), temperature (blue), and relative humidity (RH, magenta), where the oscillation of the temperature was due to the operation of a heater during winter. In this case, the PL apparatus was set at 1 m above the box with the shutter, and only a delayed (non-instantaneous) PL response was observed. On the other hand, the variation in the relative humidity as a result of the temperature change can be seen, as expected. 

To separately estimate the effect of T and RH on PL intensity, we heated a polymer substrate and measured PL, while the RH inside of the lab remained constant. In this case, the temperature of the substrate was measured with a sensor (DS18B20) which was in direct contact with the substrate.

**Figure 7:**
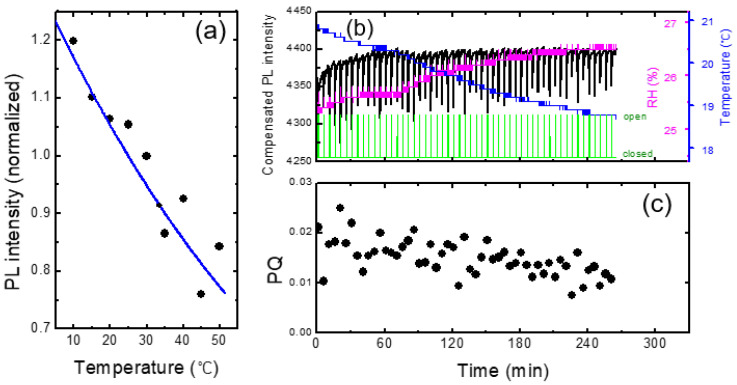
(**a**) PL intensity vs. temperature for a PCC film. In contrast to the case of Figure 6, RH remained constant as only the film substrates were heated and cooled. (**b**) PL intensity compensated for temperature variation (black) as a function of time. Temperature and RH are also shown. (**c**) PQ as a function of time.

In Figure 7a, we show the PL intensity as a function of temperature for a PCC film. Arrhenius equation I_0_/(1 + Aexp(−E_a_/k_B_T)) was used for the fitting curve, where A = 265, E_a_ = 0.131 eV, and k_B_ is the Boltzmann constant. Similar to the result in Figure 6, PL intensity was reduced by about 1% for the increase in the temperature by 1 degree. This result indicates that when the change of RH was within 2%, the effect of humidity on PL intensity was less than that of the temperature.

In Figure 7b, we show the PL intensity compensated with temperature variation using the raw data in Figure 3. The PL intensity after 60 min of the device operation remained fairly constant after the compensation, whereas the initial PL increase for the first 20 min was still clearly observed. The PL enhancement for the first 20 min was attributed to the planarization of polymer chains by light [11,35,36,37]. 

The temperature compensation appeared to be very powerful and sufficient for the monotonic and slow change of the temperature. However, for more complicated data, such compensation was not sufficient, as we will discuss in the next section.

As shown in Figure 7c, the PQ value was found to be reduced as a function of time, which was attributed to the decrease in the vapor pressure as the continuous operation of the shutter. On the other hand, even before the vapor pressure inside of the box was greatly decreased, or even when the operating time was only half an hour, the standard deviation of PQ was found to be fairly large. As the air containing the DNT vapor was released from the box when the shutter was open, it would take some time to reach the equilibrium vapor pressure outside of the box; when we measured the PQ immediately after opening the shutter, the distribution of the vapor molecules may not be uniform, possibly causing the large variation of PQ values.

**Figure 8:**
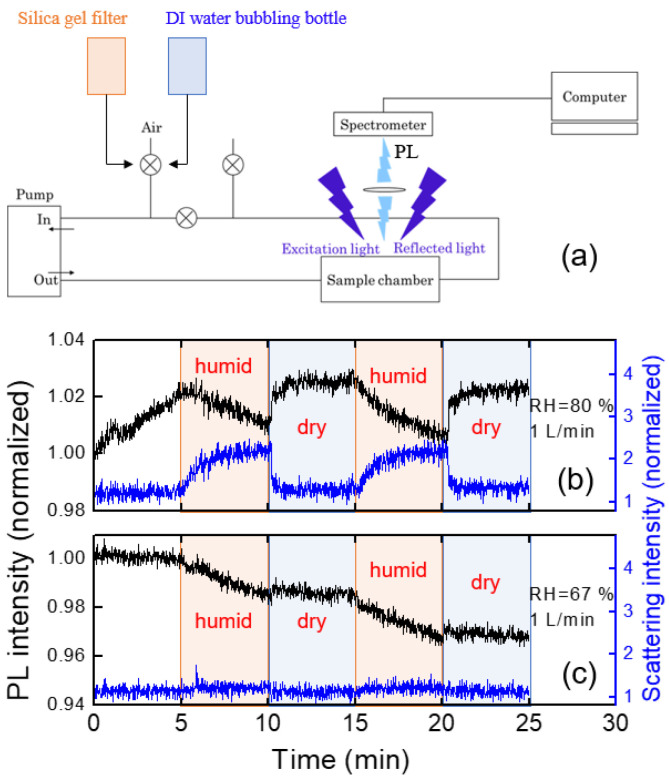
(**a**) A schematic diagram of an experimental set-up for humidity effects. (**b**,**c**) Variation of PL intensity (black) and parasitically scattered light intensity (blue) under dry and humid air conditions.

To estimate the humidity effect, we utilized a syringe filter filled with silica gel and a bottle containing DI water, and the experiments were conducted by alternating filtering and bubbling connected with our home-made PL system. A schematic diagram for the experimental setup is shown in Figure 8a. The values of RH were estimated to be around 20% (filtering) and 90% (bubbling), corresponding to the “dry” and “humid” conditions, respectively. For RH = 90%, PL intensity gradually decreased (Figure 8b), which was recovered within a second with dry air with 1 L/min flow rate. 

The humidity dependence of the PL intensity can be somewhat complicated by some additional factors: (1) under very high humid conditions, water molecules may condense on the optical components, reducing the apparent PL intensity; (2) scattering of PL in the air may be affected by the water molecules.

It was found that the immediate change of the PL intensity with dry air within tens of seconds was associated with the reduction of the scattering at the surface of optical components or at the surface of the polymer film. The parasitically scattered light was estimated from the intensity near the LED excitation wavelength of 400 nm (Figure 8b). The origin of the increased scattering under such humid air conditions may be due to the water condensation on the surface of optical components as well as of the polymer films. With the injection of the dry air, the PL intensity was quickly recovered.

We also used room air on a humid day (RH = 67%) during the rainy season in July. Figure 8c shows the PL intensity change of the polymer thin film under humid and dry air conditions. In this case, there was no abrupt change in PL intensity, unlike Figure 8b, but the PL intensity slowly decreased during the humid period. 

In the case of RH = 67%, as in Figure 8c, the parasitically scattered light remained almost constant, indicating the water condensation effect and the scattering by water molecules were minor, whereas a slow decrease in PL intensity was observed. When polymer films are exposed to humid air, the PL intensity may not be fully recovered, even after exposure to dry air due to the degradation of the polymer. 

On rainy days, a false positive detection may occur more frequently due to the light scattering of PL associated with the water condensation. In addition, the deterioration of polymer films needs to be considered after a long period of device operation, especially in very humid weather, or after the exposure of highly concentrated DNT vapors.

### 3.4. Analysis of Complicated Situations and Degradation of Polymer Films

In some cases, the compensation of temperature and humidity may be sufficient for the compensation of sensing characteristics such as PL and resistivity. However, when environmental factors are complicated, those corrections alone cannot fully explain the data. In particular, in the case of vapor phase measurement, since the vapor collection efficiency is greatly affected by the airflow, care must be taken in data processing and statistical analysis.

**Figure 9:**
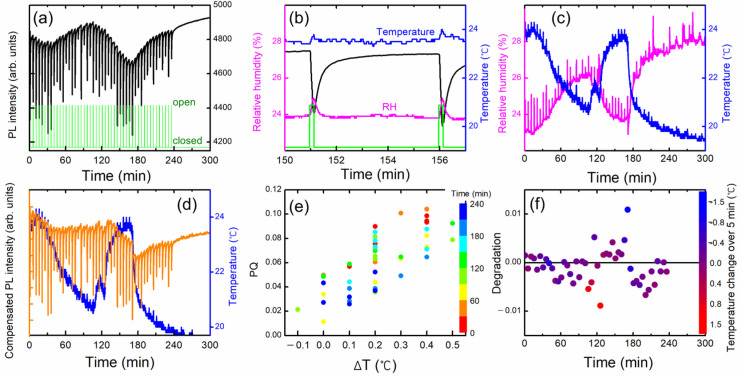
(**a**) PL intensity variation as a function of time. (**b**) An enlarged view of temperature T and relative humidity RH to demonstrate the variations under shutter operations. As the box was heated, a slight increase in the temperature ΔT during the DNT exposure time of 10 s was observed. (**c**) T and RH as a function of time. (**d**) PL intensity compensated with temperature variation. (**e**) PQ vs ΔT during the 10 s exposure time. The correlation was due to the warm airflow. The colors represent the elapsed time. (**f**) Degradation, or the incomplete recovery of PL intensity after five minutes. The color bars represent the temperature change in 5 min.

It is very challenging to understand the effect of convection wind inside a building especially when we use a heater, or when we open the windows. The distribution of the DNT vapor molecules may constantly vary with convection airflow. We examined how the convection and turbulence caused by the temperature difference affected the PL quenching results, while we heated the box by placing a heater under the acrylic box. Figure 9 shows an example of a dataset to demonstrate such complicated effects. With every shutter opening, the change of temperature (ΔT) inside the PL apparatus was monitored (Figure 9b) and compared with PQ efficiencies (Figure 9e).

When the shutter was open, not only DNT vapors but also warmer air diffused out and entered the PL apparatus, and an instantaneous increase in temperature (ΔT) was observed, which was a fairly good indicator of airflow. In some cases, the humidity inside the acrylic box was slightly higher than outside the box, possibly due to the speed difference in moisture condensation and drying. In such cases, ΔRH was also clearly observed (Figure 9c), despite the difference in the diffusion coefficients of DNT and water vapors.

In the experimental set-up, the PL apparatus was located slightly higher than the center of the shutter to optimize the PQ efficiency; the greater the upward convection, the greater the amount of DNT vapor flowing into the apparatus. The correlation between ΔT and PQ shown in Figure 9e was likely due to the airflow.

The orange curve in Figure 9d represents the PL intensity compensated for temperature variation. In this case, the value of PQ was 5.4 (±2.4)%, which was larger due to the heating of the box. For a~1 and b~0, PL intensity would completely recover within 5 min according to the desorption model. After about 130 min, however, it was found that the PL intensity was not fully recovered within 5 min after the large PL quenching. Although we compensated the PL intensity with the temperature change, PL intensity was decreased with increasing time and with continuous exposure to the high concentration of the DNT molecules. It is worth mentioning that the PL intensity seemed to recover without any shutter operation between 240 and 300 min.

To quantify the incomplete recovery of PL intensity or the degradation, we plot (I_0_ − I (5 min))/I_0_. Figure 9f shows the degradation as a function of the PQ value after the temperature compensation. As seen in Figure 9f, there were two data points (blue and red) that exhibited significant deviations. We think that these points were associated with the rapid changes in temperature and a slower response of the polymer film.

**Figure 10:**
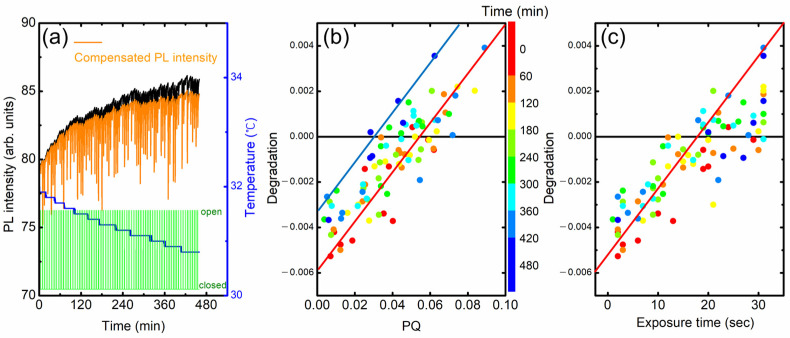
(**a**) PL intensity without (black) and with (orange) temperature compensation. (**b**) Degradation vs. PQ. The color indicates elapsed time. (**c**) Degradation as a function of exposure time.

In Figure 10a, we show the PL intensity variation of a fresh (as-cast) film. Even after the temperature compensation, PL intensity was found to still increase with time (orange). In the modeling in the previous section, we assumed that the incomplete PL recovery is proportional to PQ. In a plot of degradation vs. PQ, the slope and y-intercept are expected to be “1 − a” and “−b(t − (1 − a)t_q_)”, respectively.

In practice, these parameters depend on several factors such as the condition of the polymer film, elapsed time, and PQ value. For example, the extent of the increase appeared to be larger for as-deposited films, and the y-intercept became more negative in the degradation vs. PQ plot. Figure 10b shows the degradation as a function of PQ obtained from the raw data in Figure 10a. The color indicates elapsed time. The y-intercept was slightly increased from ~−0.005 to ~−0.0035 with the time from ~60 to ~400 min. Similarly, the degradation was also increased with the increase in the exposure time, as seen in Figure 10c. 

## 4. Conclusions

Explosives detection using PL quenching has been extensively studied for decades, but it is difficult to find reports on a statistical analysis of environmental effects. This is probably because it is difficult to obtain a sufficient amount of environmental data including temperature, humidity, and convection airflow, and it is also difficult to take care of the continuous degradation of sensing films. We implemented a method that allowed the vapor molecules inside a box to diffuse out into the air via remote control of a shutter, while the direct exposure of the pollutant vapors to a human body was prevented via remote sensing.

To obtain a sufficient amount of data without significant degradation of a sensing film, we chose a rather short exposure time (10 s). We then considered the incomplete recovery of the PL intensity after the refresh time of 5 min. The degradation, or the incomplete recovery of the PL, was more evident for higher DNT vapor pressure, longer exposure, and higher humidity.

In this study, the temperature and humidity effect in the PL quenching method using CP was discussed, and the time response due to DNT exposure was analyzed with a theoretical model. The model accounted for the case of short exposure times and relatively low vapor concentrations and provided a description of the temporal behavior of PL quenching and refreshing. Despite many assumptions and simplifications, our theoretical model seemed to well describe the experimental results, at least under limited degradation conditions. Photo-induced PL enhancement, photo-degradation, and diffusion (out-diffusion) of DNT molecules into (from) the inner part of the polymer films were taken into consideration empirically with parameters a and b in the model. 

Among environmental parameters, the effect of temperature variation on PL intensity was very significant (~1%/°C). In particular, under low vapor concentrations of explosives, the effect of temperature variation can be greater than the PL quenching effect by explosives, and the compensation of PL intensity was necessary. We demonstrated that research not only on the detection sensitivity but also on various environmental effects is important. Our study may be useful for real-field applications of PL-based chemical gas sensors and for other types of chemical sensors based on semiconductors and polymers.

## Figures and Tables

**Table 1:** Fitting parameters used for a theoretical model.

Parameter	Value	Unit
P_2,4-DNT_	1.93 × 10^−7^	
k_desorption_	4.698 × 10^−2^	s^−1^
a	8.755 × 10^−1^	
b	7.820 × 10^−6^	s^−1^
1/τk_q_k_adsorption_P_2,4-DNT_P_r_	63.61	s
k_adsorption_P_2,4-DNT_P_r_ + k_desorption_	4.701 × 10^−2^	s^−1^
(L_D_/d)tanh(d/L_D_)	1.094 × 10^−1^	

## Data Availability

Data can be provided upon request.

## Supplementary Materials

The following supporting information can be downloaded at https://www.mdpi.com/article/10.3390/polym16070908/s1. Figure S1. PL intensity with various air-flow rates plotted over time to compare the degradation of three different polymer films: PEE, MEH-PPV, and PDY-132 (super yellow).

## Dual-use Research Statement

We have made the following statements to our paper titled “Estimation of Environmental Effects and Response Time in Gas-Phase Explosives Detection Using Photoluminescence Quenching Method”.

Ø Explanation of Potential Risks: The aim of our research is to provide some theoretical and experimental understanding for vapor phase explosives detection and our research does not pose any threat to public health or national security.
Ø Evaluation of Benefits to the General Public: Our research is beneficial to the development of material science. There is no risk to the general public.
Ø Compliance with Laws: As an ethical responsibility, we strictly adhere to relevant national and international laws about dual-use research. We have considered and adhered to these regulations in our paper.
