# Supplementary material for: Estimation of Environmental Effects and Response Time in Gas-Phase Explosives Detection Using Photoluminescence Quenching Method
Source: Polymers (Basel). 2024 Mar 26;16(7):908. doi: 10.3390/polym16070908 (PMC11013195; doi:10.3390/polym16070908)
Supplement: Supplementary file 1 [file polymers-16-00908-s001.zip › polymers-2865665-supplementary.pdf]

Supplementary Materials

Estimation of environmental effects and response time in gas phase explosives detection using photoluminescence quenching method

Daegwon Noh [1,2] and Eunsoon Oh [1,2,*]

1 Department of Physics, Chungnam National University, 99 Daehakro, Yuseong-gu, Daejeon 34134, Korea
2 Institute of Quantum Systems (IQS), Chungnam National University, 99 Daehakro, Yuseong-gu, Daejeon 34134, Korea; fo1109@cnu.ac.kr (D.N.)
* Correspondence: esoh@cnu.ac.kr.

Supplementary figure 1 shows the normalized PL intensity of PEE, MEH-PPV, and PDY-132 (super yellow) films as a function of time. PEE, MEH-PPV, and PDY-132 powders were purchased from Sigma-Aldrich and the experimental conditions were all identical for the three films. In this measurement, the flow rate was controlled with a pump [11]. The pump was turned on and off for a minute for each flow rate. Blue shaded parts indicate the pump-on period. The flow rate was changed from 1 L/min to 6 L/min. The cycle was repeated 3 times. As demonstrated in this paper, the PL intensity of PCC (pentiptycene-containing conjugated polymer) film did not decrease noticeably even after several hours of continuous measurement in contrast to the cases of MEH-PPV and PDY-132.

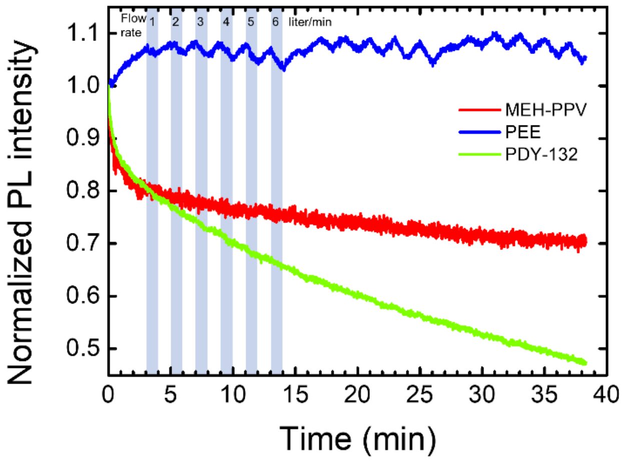

Figure S1. PL intensity with various air-flow rates plotted over time to compare the degradation of three different polymer films: PEE, MEH-PPV, and PDY-132 (super yellow).

Reference [11]: Noh, D.; Ampadu, E. K.; Oh, E. Influence of Air Flow on Luminescence Quenching in Polymer Films towards Explosives Detection Using Drones. *Polymers* **2022**, *14* (3), 483. https://doi.org/10.3390/polym14030483.
